# Correction: EPO Promotes Bone Repair through Enhanced Cartilaginous Callus Formation and Angiogenesis

**DOI:** 10.1371/journal.pone.0111830

**Published:** 2014-10-22

**Authors:** The PLOS ONE Staff

## Notice of Republication

This article was republished on September 23, 2014, to correct the publication year in the footer of the PDF. The publisher apologizes for these errors. The originally published, uncorrected article and the republished, corrected article are provided here for reference.

## Supporting Information

File S1 Originally published, uncorrected article
(PDF)

File S2 Republished, corrected article
(PDF)
