# Supplementary material for: Correction: EPO Promotes Bone Repair through Enhanced Cartilaginous Callus Formation and Angiogenesis
Source: PLoS One. 2014 Oct 22;9(10):e111830. doi: 10.1371/journal.pone.0111830 (PMC4206514; doi:10.1371/journal.pone.0111830)
Supplement: File S1 — Originally published, uncorrected article (PDF) [file pone.0111830.s001.pdf]

EPO Promotes Bone Repair through Enhanced Cartilaginous Callus Formation and Angiogenesis

Lin Wan[1], Fengjie Zhang[1,2], Qiling He[3], Wing Pui Tsang[1,2], Li Lu[1,4], Qingnan Li[4], Zhihong Wu[5], Guixing Qiu[5], Guangqian Zhou[6], Chao Wan[1,2]*

1 Ministry of Education Key Laboratory for Regenerative Medicine, School of Biomedical Sciences, Faculty of Medicine, The Chinese University of Hong Kong, Hong Kong SAR, China, **2** School of Biomedical Sciences Core Laboratory, Shenzhen Research Institute, The Chinese University of Hong Kong, Shenzhen, China, **3** Departments of Microbiology and Pathology, University of Alabama at Birmingham, Birmingham, Alabama, United States of America, **4** Guangdong Key Laboratory of Pharmaceutical Bioactive Substances, New Drug Function Research Center, School of Life Science and Biopharmacy, Guangdong Pharmaceutical University, Guangzhou, China, **5** Department of Orthopaedics, Peking Union Medical College Hospital, Peking Union Medical College and Chinese Academy of Medical Sciences, Beijing, China, **6** The Center for Anti-Ageing and Regenerative Medicine, Medical School, Shenzhen University, Shenzhen, China

Abstract

Erythropoietin (EPO)/erythropoietin receptor (EPOR) signaling is involved in the development and regeneration of several non-hematopoietic tissues including the skeleton. EPO is identified as a downstream target of the hypoxia inducible factor-α (HIF-α) pathway. It is shown that EPO exerts a positive role in bone repair, however, the underlying cellular and molecular mechanisms remain unclear. In the present study we show that EPO and EPOR are expressed in the proliferating, pre-hypertrophic and hypertrophic zone of the developing mouse growth plates as well as in the cartilaginous callus of the healing bone. The proliferation rate of chondrocytes is increased under EPO treatment, while this effect is decreased following siRNA mediated knockdown of EPOR in chondrocytes. EPO treatment increases biosynthesis of proteoglycan, accompanied by up-regulation of chondrogenic marker genes including SOX9, SOX5, SOX6, collagen type 2, and aggrecan. The effects are inhibited by knockdown of EPOR. Blockage of the endogenous EPO in chondrocytes also impaired the chondrogenic differentiation. In addition, EPO promotes metatarsal endothelial sprouting *in vitro*. This coincides with the *in vivo* data that local delivery of EPO increases vascularity at the mid-stage of bone healing (day 14). In a mouse femoral fracture model, EPO promotes cartilaginous callus formation at days 7 and 14, and enhances bone healing at day 28 indexed by improved X-ray score and micro-CT analysis of microstructure of new bone regenerates, which results in improved biomechanical properties. Our results indicate that EPO enhances chondrogenic and angiogenic responses during bone repair. EPO's function on chondrocyte proliferation and differentiation is at least partially mediated by its receptor EPOR. EPO may serve as a therapeutic agent to facilitate skeletal regeneration.

Citation: Wan L, Zhang F, He Q, Tsang WP, Lu L, et al. (2014) EPO Promotes Bone Repair through Enhanced Cartilaginous Callus Formation and Angiogenesis. PLoS ONE 9(7): e102010. doi:10.1371/journal.pone.0102010

Editor: Domenico Ribatti, University of Bari Medical School, Italy

Received April 18, 2014; **Accepted** June 13, 2014; **Published** July 8, 2014

Copyright: © 2014 Wan et al. This is an open-access article distributed under the terms of the Creative Commons Attribution License, which permits unrestricted use, distribution, and reproduction in any medium, provided the original author and source are credited.

Data Availability: The authors confirm that all data underlying the findings are fully available without restriction. All relevant data are within the paper and its Supporting Information files.

Funding: This work was supported by Hong Kong Research Grant Council General Research Fund (RGC GRF 475910), National Natural Science Foundation of China (NSFC 81130034), and NSFC-RGC Joint Research Scheme (CUHK445/10) to CW. The funders had no role in study design, data collection and analysis, decision to publish, or preparation of the manuscript.

Competing Interests: The authors have declared that no competing interests exist.

* Email: cwan@cuhk.edu.hk.

Introduction

Impaired bone regeneration following injury or under pathological conditions causes severe pain to the patients and considerable financial burden to the society [1,2]. These conditions include delayed fracture union or non-union, osteoporotic fracture healing, impaired bone repair associated with diabetes, and large bone defects caused by trauma or surgical treatments [3,4,5,6]. Hallmarks of impaired bone repair in patients and animal models include the deficiency in vascular supply and cartilaginous callus formation at the site of injury, suggesting that impaired angiogenic and chondrogenic responses are major contributors to the pathology [7,8].

Bone regeneration is a complex process in which the recovery of skeletal tissue integrity relies upon close temporal and spatial coordination of molecular and cellular events involving resident bone cells, inflammatory cells, marrow stromal elements, and associated vascular structures [9,10,11,12] to achieve structural reconstitution and bone remodeling. The regeneration process has generally been characterized by four steps including inflammation phase with hematoma, cartilage callus formation, bony callus formation and bone remodeling [13,14]. During bone regeneration, angiogenesis is essential and depends on hypoxic stimuli and production of proangiogenic factors such as vascular endothelial growth factor (VEGF) [7], Erythropoietin (EPO) [15], fibroblast growth factor (FGF) [16], transforming growth factor-beta (TGF-beta) [17] and insulin like growth factor (IGF-1) [18].

The bone cells are readily located in a hypoxic microenvironment during development and regeneration. Hypoxia inducible factor-α (HIF-α) is identified as key mediator for cell adaptation to low oxygen tension. VEGF and EPO are established downstream targets of HIF-α. It is well established that VEGF is expressed in

osteoblasts [19,20] and the HIF-α/VEGF pathway couples angiogenesis and osteogenesis during bone development and regeneration [7,21,22] while the study on the involvement of EPO in bone regeneration is just emerging.

EPO is a 34 kD circulating glycoprotein hormone which is initially recognized as the crucial growth factor that controls red blood cell development in bone marrow through binding to its high affinity receptor (EPOR) expressed on erythroid progenitor cells [23,24,25]. EPO is predominantly produced by the interstitial fibroblasts of the renal cortex and outer medulla in adult animals, and by Kuffer's cells in the liver during embryonic development [26,27,28,29], under the control of oxygen sensing HIF pathway [30]. In addition to its principal function in erythropoiesis, nonehematopoietic functions of EPO such as angiogenic [25], cardioprotective [31], neuroprotctive and neurotrophic [32,33] effects have raised great interest and been extensively studied, due to the findings that EPO is also produced in non-renal tissues such as brain [34], and that EPOR is present in a variety of non-hematopoietic cell types such as brain capillary endothelial cells [35], vascular smooth muscle cells [36], myocardial cells [37], neurons [38] and astrocytes [39]. The potential physiological effects of EPO on skeletal tissue were suggested by both clinical and animal studies that bone modulation was associated with erythropoiesis stimulation by systemic administration of EPO [40,41,42]. However, the underlying mechanism is largely unknown. It was reported that EPO activated JAK/STAT signaling in hematopoietic stem cells (HSCs) to produce bone morphogenetic proteins (BMPs) and thus exerted indirect function in bone formation [43]. Although there is one study showing that EPO indirectly increased bone turnover rate and induced trabecular bone loss [42], several other studies demonstrated that EPO improved bone healing [44,45,46], possibly through the proposed direct interaction between EPO with EPOR-expressed hypertrophic chondrocytes to promote endochondral ossification [45] or with EPOR-expressed bone marrow stromal cells to induce osteoblastic differentiation [43]. In addition, significant vascular expansion was also noted during bone healing when EPO was applied [44,46]. It is known that EPO and EPOR are expressed in the vasculature during embryogenesis, and exert a critical role for angiogenesis [25]. Therefore the improved bone healing may also be attributed to a better angiogenesis caused by EPO.

In the present study, we systemically examined the roles of EPO in regulating chondrogenesis and angiogenesis *in vitro* and *in vivo*. The promotive effects of EPO during the chondrogenic and angiogenic phases of femur fracture repair highlight its therapeutic potential in skeletal regenerative medicine.

Materials and Methods

Animals

C57BL/6 mice at age of three-days, fourteen-days and twelve-weeks were used in this study. Mice were housed in the controlled conditions with room temperature of 22±1°C and a light-dark cycle of 12:12 hour, and were fed with standard mouse chow and water ad libitum. Experimental procedures were carried out in accordance with the protocols approved by Animal Experimentation Ethics Committee from the Chinese University of Hong Kong and Animal (Control of Experiments) Ordinance from Department of Health, Hong Kong SAR.

Culture of primary chondrocytes from neonatal mice

The primary chondrocytes were isolated from growth plates following previous procedures [47]. Cells were cultured in minimum essential medium alpha (αMEM) supplemented with 10% fetal bovine serum (FBS), 2 mM L-glutamine and 100 U/ml penicillin and 100 μg/ml streptomycin (all from Gibco). The 1st and 2nd passage cells were processed in the below experiments.

Knockdown of EPOR in chondrocytes

Knockdown of EPOR in chondrocytes was performed by siRNA (Santa Cruz) method using lipofectamine2000 (Invitrogen) following standard protocol. 24 hours after transfection, cells were collected for real-time PCR and Western blot analysis to examine the efficiency of gene knockdown.

Bromodeoxyuridine (BrdU) incorporation assay

Chondrocytes were treated with 40 ng/ml or 80 ng/ml EPO (Sigma-Aldrich) for 2 days and compared with that without EPO treatment. The proliferation rate of chondrocytes was analyzed using the cell proliferation ELISA BrdU assay (Roche).

Cell mass culture

Cells were plated at density of 1×10^5 cells/10 μl and cultured for 3 hours to form the cell masses. Cell masses were then treated with no EPO, 40 ng/ml EPO or EPO block peptide (500 ng/ml, Santa Cruz) in chondrogenic medium supplemented with dexamethasone (100 nM, Sigma-Aldrich), ascorbic acid (50 μg/ml, Sigma-Aldrich), sodium pyruvate (100 μg/ml, Gibco), ITS+ Premix (50 mg/ml, BD) for 7 days following previous procedures [48].

Alcian blue staining

The Alcian blue staining was performed to examine proteoglycan synthesis during chondrocytes differentiation as previously described [49]. Briefly, the cell masses were rinsed with PBS after 7 days of culture, and stained with 1% acidic Alcian blue for 2 hours, followed by 5 times washing using 70% ethanol to remove the residual dyes. The proteoglycan was stained blue.

Quantitation of sulfated glycosaminoglycan (sGAG)

sGAG was quantitated using a dimethylmethylene blue colourimetric assay following established protocol (Blyscan sulfated glycosaminoglycan assay) [50,51]. DMB dye (1, 9-Dimethyl-Methylene Blue zinc chloride double salt) was added to measure the sGAG content in the digested substances.

RT PCR and real-time PCR

Total mRNA was extracted from primary chondrocytes using the TRIzol reagent (Life Technologies) following the manufacturer's instructions. First strand cDNA was synthesized from 1 μg of total RNA in the presence of oligo-dT$_{12-18}$ primer (Invitrogen) and MMLV reverse transcriptase according to manufacturer's instructions (Promega). Quantitative real-time PCR was performed with SYBR Premix Ex Taq (Takara) in ABI Fast Real-time PCR 7900HT System (Applied Biosystems). All samples were performed in triplicates. Primer sequences used for RT-PCR include: EPO-F, 5′-GCTGCTTTTACTCTCCTTGC-3′, EPO-R, 5′-CTGTTCTTCCACCTCCATTC-3′; EPOR-F, 5′-CTCACCTTCCAGCTTT-GAGT-3′, EPOR-R, 5′-TTCTCATAGGGGTGGGAGTA-3′; β-actin-F, 5′-GTCCCT-CACCCTCCCAAAAG-3′, β-actin-R, 5′-GCTGCCTCAACACCTCAACCC-3′. Primer sequences used for real-time PCR are: SOX9-F, 5′-AGGA-AGCTGGCAGACCAGTA-3′, SOX9-R, 5′-TCCACGAAGGGTCTCTTCTC; SOX5-F, 5′-CCAGGAGCTTGTCTTTCCAG-3′, SOX5-R, 5′-CCCTGAAGCAGAGGAAGATG-3′; SOX6-F, 5′-CTTCCTCTCCATC-CGCTGTA-3′, SOX6-R, 5′-ACCAGTGACTTCTGGGTGCT-3′; aggrecan-F,

5′-CTG-AAGTTCTTGGAGGAGCG-3′, aggrecan-R, 5′-CGC-TCAGTGAGTTG-TCATGG-3′; Col2α1-F, 5′-CTACGGTGT-CAGGGCCAG-3′, Col2α1-R, 5′-GCAAGA-TGAGGGCTTC-CATA-3′; EPOR-F, 5′-AAACTCAGGGTGCCCCTCTGGC-CT-3′, EPOR-R, 5′-GATGCGGTGATAGCGAGGAGAACC-3′; β-actin-F, 5′-CCCAGAGC-AAGAGAGG-3′, β-actin-R, 5′-GTCCAGACGCAGGATG-3′.

Western blot analysis

Cells were lysed and homogenized in RIPA lyses buffer with protease inhibitors (Gibco). Aliquots of protein extracts were separated by SDS-PAGE, and blotted on PVDF membrane (Millipore). The membrane was blocked by TBS-0.1% Tween 20 (TBST) containing 5% skim milk at room temperature for 1 hour, probed with anti-EPOR (Santa Cruz) primary antibody at 4°C overnight, washed in TBST, and then incubated with the appropriate horseradish peroxidase (HRP)-conjugated secondary antibody at room temperature for 1 hour. After intensive washing with TBST, immunoblots were developed using the ECL detection system.

In vitro fetal mouse metatarsal angiogenesis assay

The assay was performed according to established procedure [52,53]. Briefly, metatarsal bones were dissected from E17.5 embryos, cultured in 24-well plates in 150 μl αMEM containing 10% fetal calf serum, L-glutamine (2 mM) and penicillin (100 U/ml)/streptomycin (100 μg/ml) for 72 hours, and then changed to 250 μl of fresh medium. Medium was then replaced every two days. After 14 days, the explants were fixed in zinc formalin for 15 minutes and subsequently immunostained for endothelial marker CD31 using a rat polyclonal antiserum (BD Pharmingen).

Femur fracture model in mice

12-week-old mice were anesthetized by intraperitoneal injection of 0.1 ml xylene (2.6 mg/ml) and ketamine (17.4 mg/ml) cocktail. A 2 cm incision was performed between the greater trochanter and the knee joint. The quadriceps femoris muscles were mobilized anteriorly towards the knee and the hip. A hole was drilled on the intracondylar notch using a 21 gauge needle which was inserted into the intramedullary canal, and then pulled out. Subsequently, an osteotomy of the mid-shaft of the femur was made by a surgical saw and the fracture was stabilized by a needle inserting into the intramedullary cavity. The muscles and skin were closed with interrupted sutures. EPO (40 ng/mouse) was administered every other day directly at the fracture site from day 4 to day 12 post-surgery, with saline as non-treatment control.

Histology, histochemistry and immunohistochemistry

Paraffin sections of the long bones were dewaxed and processed for Safranin O staining. Briefly, the sections were stained with Weigert's iron hematoxylin working solution for 5 min, washed in running tap water for 10 min, and then stained with 0.05% fast green solution for 5 min. After quick rinse with 0.1% acetic acid solution for 2 min, the sections were stained in 0.1% Safranin O solution for 5 min (materials are all from Sigma-Aldrich) followed by rapid dehydration in graded ethanol, clearness and mounting in Permount mounting medium (SP15-100, Fisher Scientific). For preparation of cryosections, the femurs were fixed in 4% paraformaldhyde for 3 days at 4°C, and decalcified in 10% EDTA for 14 days at 4°Cwith gentle agitation followed by dehydration for cryoprotection in graded concentrations (15% and then 30%) of sucrose at 4°C until the femurs were fully sank in the sucrose. The femurs were then embedded in optimal cutting temperature (OCT) compound (Tissue-Tek, Sakura Finetek USA) and flash frozen in liquid nitrogen. 5 μm thick sections were cut on a cryostat. Immunostaining for EPO and EPOR was performed following established protocols.

MicroCT angiography analysis

At 14 days post-fracture, mice were anesthetized following above procedures. The abdominal cavity and heart were exposed. After opening the hepatic portal vein, the entire vascular system was completely flushed through injection of heparinized saline (20 U/ml, Sigma-Aldrich) into the left ventricle. Microfil (Flow Tech) was prepared immediately and injected into left ventricle. The specimens were then placed at 4°C overnight before the fractured femurs were harvested. The femurs were fixed in 10% formalin for 7 days, decalcified in 10% EDTA for 14 days with gentle agitation and were then processed for micro-CT scanning. Histomorphometric parameters of blood vessels including vessel volume (VV), total volume (TV), VV/TV, vessel surface (VS), vessel thickness (VTh), vessel separation (VSp), and vessel number (VN) were analyzed.

Radiological analysis of bone

The healing bones are examined by bench top digital X-ray system at day 28 post-surgery. The bone union at fracture site was quantified according to the X-ray scoring system described by Lane and Sandhu with modifications [54]. For analysis of the 3D microarchitecture of the healing bone, the femurs were scanned by the Scanco μCT40 desktop cone-beam micro-CT scanner [55]. In brief, the femurs were placed vertically in the scanning holder with diameter of 12 μm. The scans were performed at the following settings: 12 mm resolution, 55 kvp, 145 μA with an integration time of 200 ms. Images were automatically reconstructed into 2-D slices, and region of interest was outlined in every slice via micro-μCT Evaluation Program (v5.0A, Scanco Medical). Direct calculation of bone histomorphometric parameters is performed including bone volume (BV), total volume (TV), BV/TV, bone surface (BS), trabecular thickness (TbTh), trabecular separation (TbSp) and trabecular number (TbN).

Biomechanical test

Femurs were collected at 28 days post-fracture and fresh frozen. Specimens were thawed in PBS at room temperature for 3 h before mechanical testing. Specimens were tested to failure by three-point bending using the LR10K Plus Extended machine (Lloyd Instruments Ltd). Briefly, femurs were placed horizontally on two support rollers with a span of 7 mm while a vertical load was applied to the center of the diaphysis using a material testing apparatus until failure. Biomechanical parameters including peak load, elastic modulus, bend strain at maximum and bend strength at maximum were calculated from the force displacement data.

Statistics

For the quantitative real-time PCR, cell proliferation and colony formation assays, all the experiments were repeated at least three times unless otherwise stated. Data was presented as mean ± standard deviation (SD). Statistical analysis was performed using SPSS software. Two-tailed Student's t test, one-way ANOVA or two-way ANOVA were employed according to the experimental design. $P<0.05$ was considered as statistical significance.

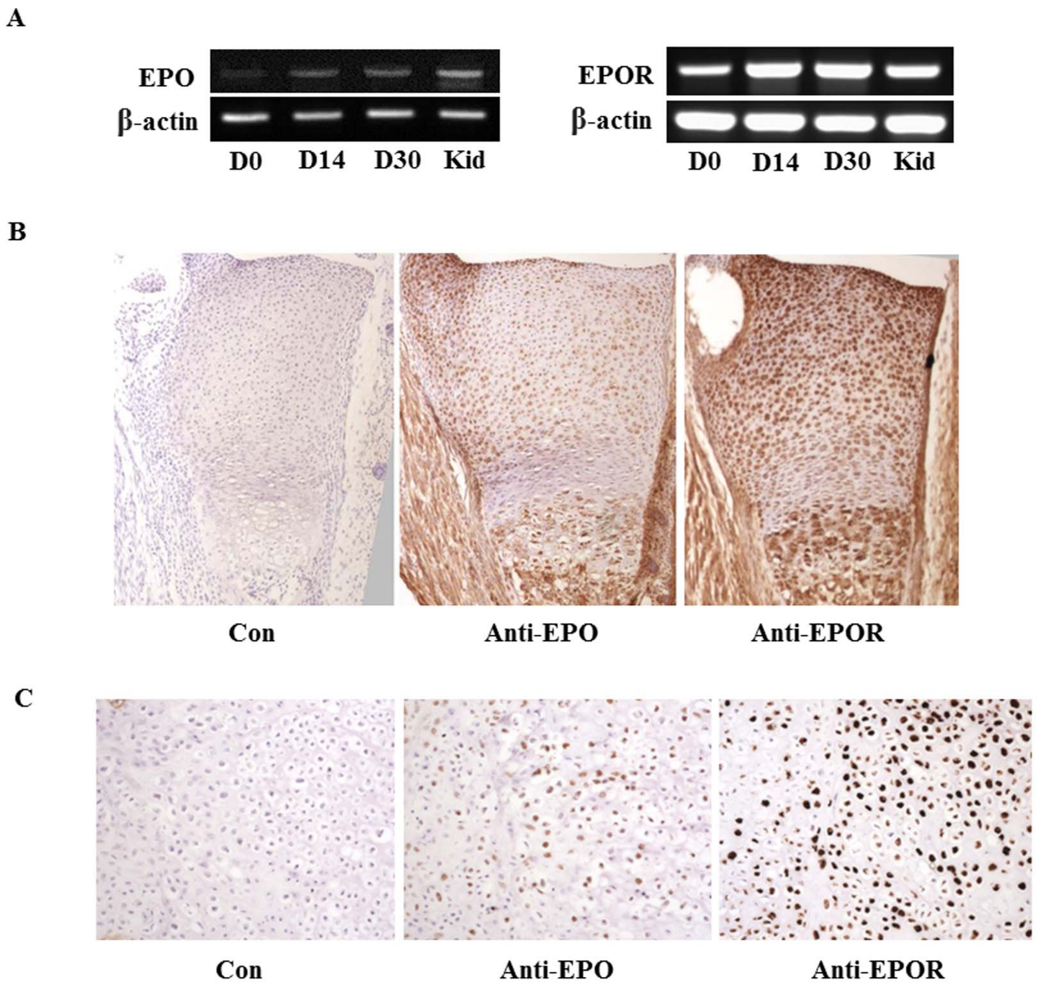

Figure 1. The expression and localization of EPO and EPOR in the developing bones. (A) mRNA expression of EPO and EPOR in the developing growth plates of tibiae by RT-PCR at indicated postnatal days. Tissues from kidney (Kid) of new born mice were used as positive control. (B) Immunohistochemistry for EPO and EPOR in growth plates of tibiae from new born mice. Con, non-primary antibody control. Magnification: 40×. (C) Immunohistochemistry for EPO and EPOR in cartilaginous callus of the bone healing in femoral fracture model of mouse. Con, non-primary antibody control. Magnification: 100×.
doi:10.1371/journal.pone.0102010.g001

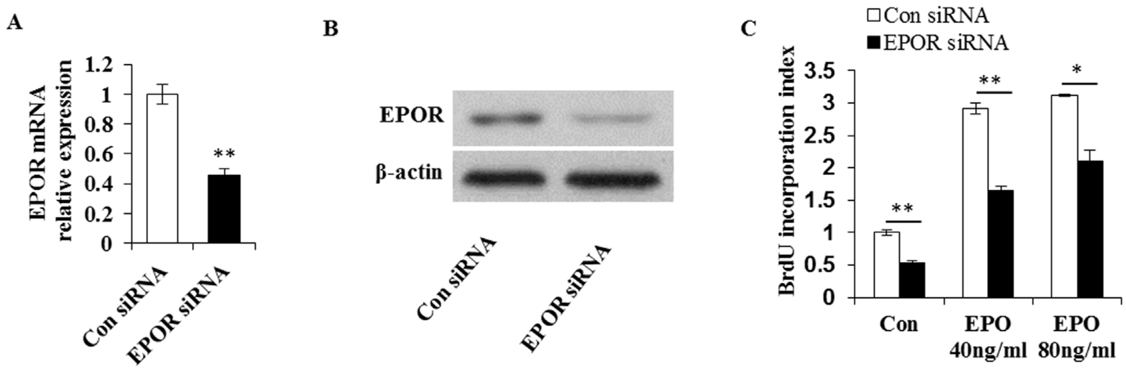

Figure 2. EPO promotes the proliferation of primary chondrocytes. (A) Examination of mRNA expression of EPOR in chondrocytes by real-time PCR after siRNA mediated knockdown of EPOR. (B) Western blot analysis of EPOR expression in primary chondrocytes with or without siRNA mediated knockdown of EPOR. (C) BrdU incorporation assay in primary chondrocytes with or without siRNA mediated knockdown of EPOR after 48 hours of EPO exposure. Con, non-treatment control. *$P<0.05$; **$P<0.01$; $n=3$.
doi:10.1371/journal.pone.0102010.g002

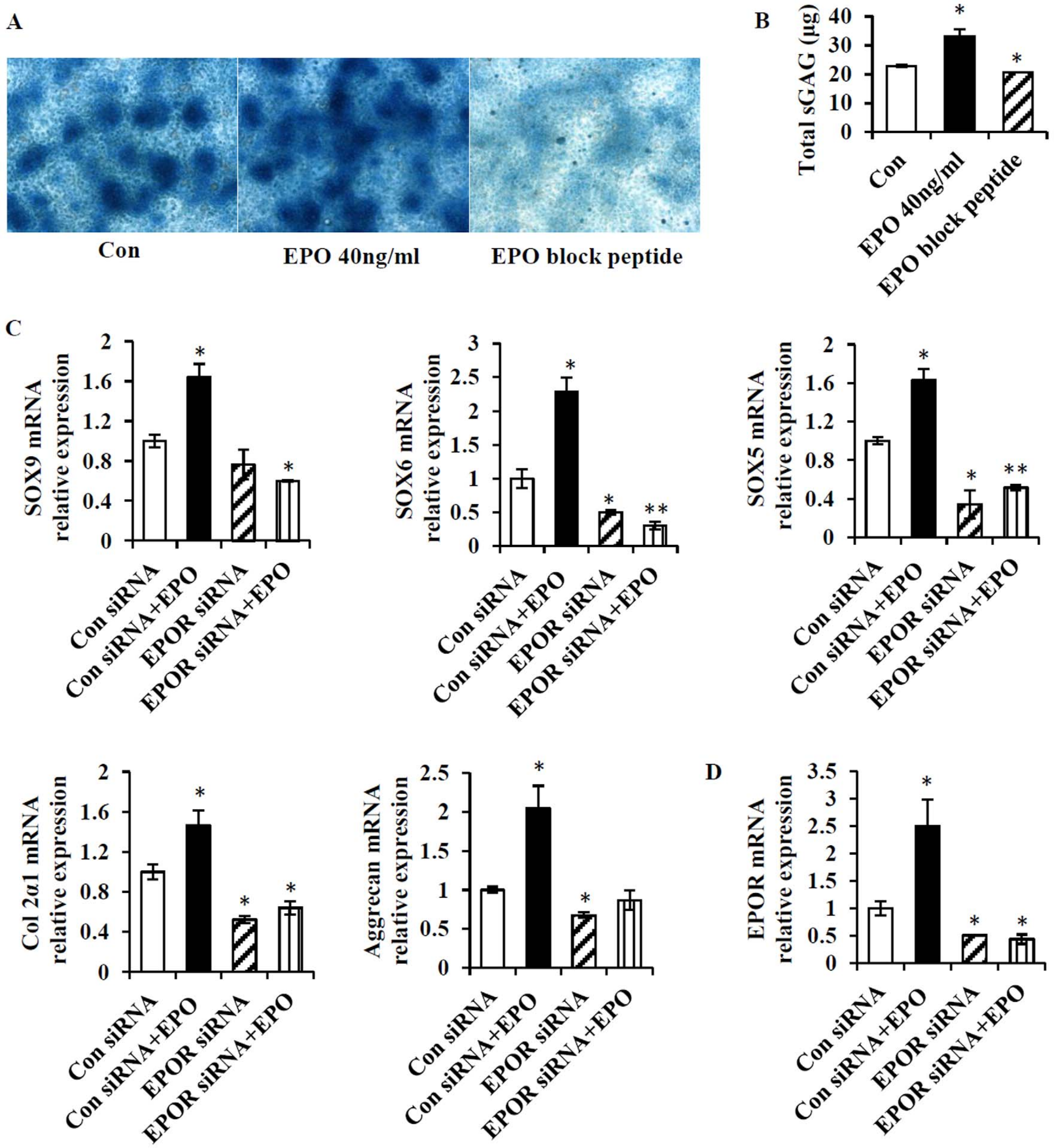

Figure 3. EPO increases biosynthesis of proteoglycan and upregulates chondrogenic marker genes expression during chondrogenic differentiation. (A) Alcian blue staining for preoteoglycan synthesis in the chondrogenic micromass culture supplemented with EPO, and with or without EPO block peptide for 7 days. Con, non-treatment control. Magnification: 10×. (B) Quantitation of total sGAG in the micromass culture by dimethylmethylene blue assay. (C) Detection of mRNA expression of chondrogenic marker genes including SOX9, SOX5, SOX6, Col2α1 and aggrecan by real-time PCR in chondrocytes in response to EPO treatment following siRNA mediated EPOR knockdown. (D) Real-time PCR quantification of EPOR mRNA expression in chondrocytes in response to EPO treatment following siRNA mediated EPOR knockdown. Con siRNA, control siRNA. *$P<0.05$; **$P<0.01$, $n=3$.
doi:10.1371/journal.pone.0102010.g003

Results

The expression and localization of EPO and EPOR in the developing growth plate and the cartilaginous callus of healing bone

To examine the expression of EPO and EPOR in chondrocytes of the developing growth plates, we performed RT-PCR for EPO and EPOR using tissues obtained from the developing long bone growth plates of new born mice. EPO and EPOR mRNA was expressed in the growth plates derived from new born mice. Both of their expression in the growth plates was also detected at ages of day 14 and 30 (Fig. 1A). To identify the localization of EPO and EPOR in the growth plates, immunostaining for EPO and EPOR was performed in new borne mice. Our results showed that EPO and EPOR were abundantly present in proliferating, pre-hypertrophic and hypertrophic zones of growth plates of the developing bones (Fig. 1B). We next examined the localization of EPO and EPOR in the cartilaginous callus in the middle stage of bone healing. It showed that Both EPO and EPOR were localized in the proliferating, pre-hypertrophic and hypertrophic chondrocytes of the cartilaginous callus (Fig. 1C). These results suggested

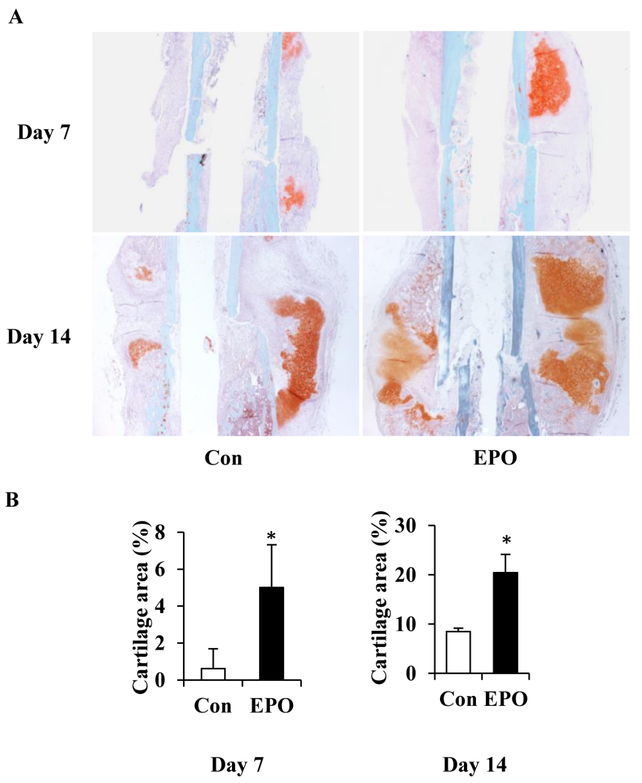

Figure 4. EPO enhances cartilaginous callus formation during bone healing. (A) Safranin O staining of the cartilaginous callus at days 7 and 14 post-surgery. Con, non-treatment control. Magnification: 2.5×. (B) Quantitation of the volume of cartilaginous callus formation at days 7 and 14 post-surgery. *$P<0.05$, $n=5$.
doi:10.1371/journal.pone.0102010.g004

that EPO and EPOR might play an important role in the physiological development of the growth plate and cartilaginous formation during bone healing.

EPO increases the proliferation of primary chondrocytes

To elucidate EPO function in the development of growth plate, we first determined the effect of EPO treatment on the proliferation of primary chondrocytes with or without knockdown of EPOR. Using siRNA method, we achieved approximately 60% knockdown efficiency in the EPOR transcription level as shown by quantitative real-time PCR (Fig. 2A). Western blot verified the dramatically down-regulated EPOR protein level in chondrocytes following EPOR-siRNA transfection (Fig. 2B). We then added varied doses of EPO into the cultures of the primary chondrocytes transfected with control-siRNA or EPOR-siRNA, and cultured for two days followed by 6 hours of BrdU pulse labeling. In the cultures of the control-siRNA transfected chondrocytes, the BrdU incorporation index (indicating the numbers of BrdU incorporated cells) increased by (1.91±0.04) units in the 40 ng/ml EPO treatment group compared with that in the untreated group, and further increased by (2.12±0.02) units when 80 ng/ml EPO was applied (Fig. 2C), indicating that EPO may serve as a mitogenic factor for primary chondrocytes. We next asked whether the EPO-induced increase in the chondrocyte proliferation was EPOR dependent by looking at the effect of EPO on the chondrocytes following EPOR knockdown. Indeed, EPO's mitogenic effect on primary chondrocytes significantly decreased following siRNA mediated knockdown of EPOR. The increase of BrdU incorporation index induced by 40 ng/ml EPO and 80 ng/ml EPO dropped to (1.11±0.11) units and (1.57±0.08) units respectively in the chondrocytes transfected with EPOR-siRNA, which was significantly lower than what was observed in the control-siRNA groups (1.11 vs 1.91 and 1.57 vs 2.12; $P<0.05$). These results suggested that at least part of the proliferative effect of EPO on chondrocyte was mediated by its cognate receptor EPOR. Whether the elevation in BrdU incorporation index in EPOR-knockdown groups induced by the addition of EPO was caused by the interaction of EPO with the remaining EPOR, or by EPOR-independent function of EPO remains to be defined. Of interest, after knockdown of EPOR, the number of BrdU incorporated chondrocytes was significantly reduced when the cells were cultured in basal medium without exogenous EPO (Fig. 2C), suggesting that EPOR may be involved in the chondrocyte proliferation either independently or through its interaction with the endogenous EPO present in the chondrocytes.

EPO promotes chondrogenic differentiation *in vitro*

We then examined whether EPO also regulates chondrogenic differentiation by monitoring the proteoglycan biosynthesis in the extracellular matrix of chondrocyte micromass cultures following chondrogenic induction. After 7 days of chondrogenic induction. EPO (40 ng/ml) administration group showed more intense Alcian blue staining of chondrogenic nodules than the control group without EPO treatment (Fig. 3A), indicating increased amount of proteoglycan production. However, there appeared fewer and smaller Alcian blue stained nodules in the group where endogenous EPO was blocked by using EPO block peptide (Fig. 3A). The total amount of sGAG in chondrocytes was increased by 40% under EPO administration compared with the control group without EPO treatment, while there was a 20% decrease in the group treated with EPO block peptide (Fig. 3B). The reduction in proteoglycan levels after blockage of endogenous EPO and the increase of them following the addition of exogenous EPO implied the significance of both endogenous and exogenous EPO in chondrogenic differentiation.

To further understand the function of EPO in chondrogenic differentiation, mRNA expression of chondrogenic marker genes including SOX9, SOX5, SOX6, aggrecan, and Col2α1 was evaluated by quantitative real-time PCR in the micromass cultures of the EPOR-knockdown and control chondrocytes following 7 days of EPO treatment. The SOX family members SOX9, SOX5 and SOX6 have been demonstrated to be key transcription factors for chondrocyte specification [56,57]. EPO administration significantly up-regulated the above chondrogenic marker genes in the control chondrocytes by 1.5 to 2.5-fold while dramatic down-regulation of these marker genes was observed in EPOR-knockdown chondrocytes without EPO treatment (Fig. 3C), moreover, addition of EPO to the EPOR-knockdown chondrocyte cultures did not increase the expression of these genes to the levels that were comparable to what was achieved in the control chondrocytes treated with EPO (Fig. 3C). In addition, exogenous EPO significantly up-regulated EPOR mRNA level in chondrocytes (Fig. 3D). These results clearly demonstrated that EPO exerted a positive effect on chondrogenic differentiation, and this effect was mediated at least partially by EPOR.

EPO enhances cartilaginous callus formation during bone healing

We next determined the role of EPO in chondrogenesis *in vivo* using a femur fracture model in mouse. Direct delivery of EPO at

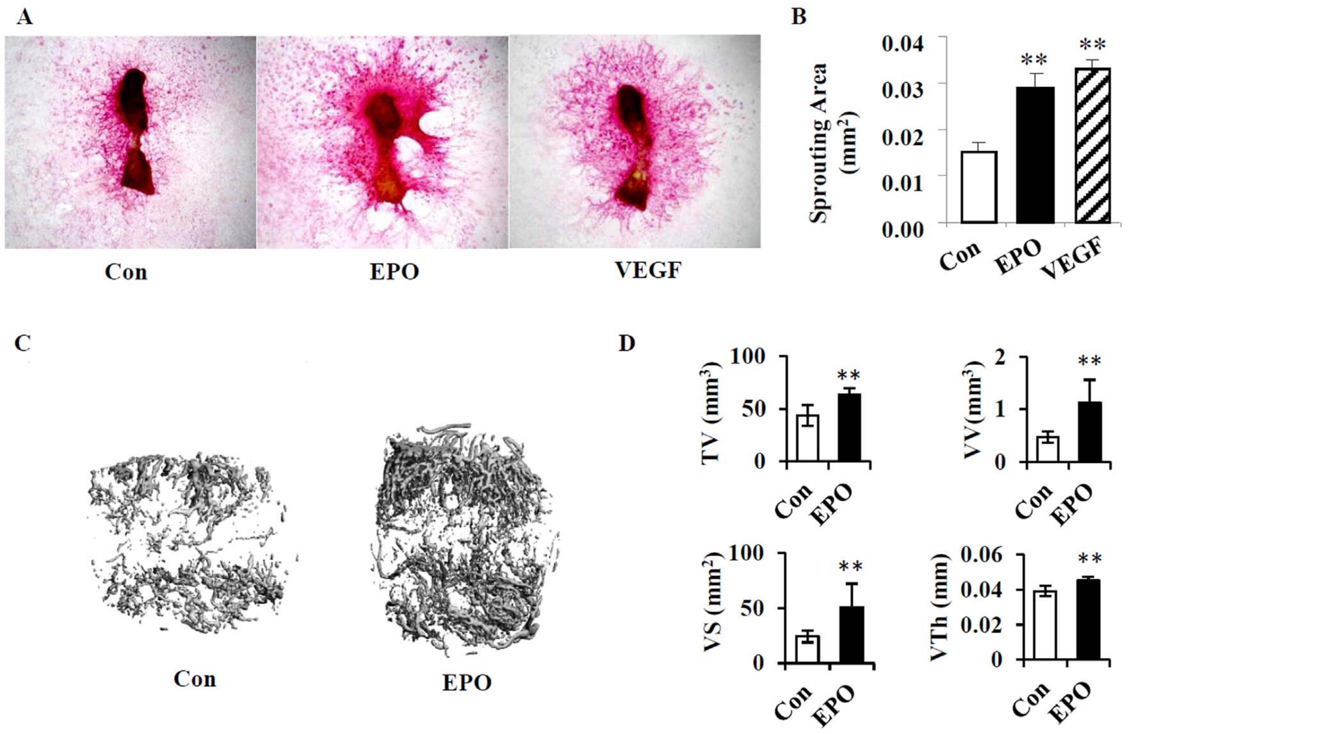

Figure 5. EPO stimulates angiogenesis *in vitro* and *in vivo*. (A) Representative images of endothelial sprouting from metatarsal bones of E17.5 embryos which were treated with different conditions as indicated. The endothelial sprouting was visualized by immunostaining for CD31. VEGF was used as positive control. Con, non-treatment control. Magnification: 2.5×. (B) Quantitation of the endothelial sprouting area for each group. (C) Representative micro-CT 3D reconstruction of vasculature at the fracture site following EPO treatment. The specimens were harvested for micro-CT scanning at day 14 post-surgery. Con, non-treatment control. (D) Quantitation of vascular parameters including the total volume (TV), vessel volume (VV), vessel surface (VS), and vessel thickness (VTh) by micro-CT angiography analysis. ** $P<0.01$, $n=5$.
doi:10.1371/journal.pone.0102010.g005

the fracture site was performed every other day from day 4 to day 12 post-surgery. At days 7 and day 14 post-surgery, cartilaginous callus formed at the fracture sites was visualized by Safranin O staining (Fig. 4A). Histomorphometry analysis of cartilaginous callus of the healing bones showed that callus size and cartilage area in the EPO treatment group were larger than that in the saline control group (Fig. 4A). Upon EPO treatment, the ratios of cartilage volume to the total tissue volume were 5% and 20% respectively at day 7 and day 14 post-surgery, while the saline control group only gave rise to the ratios of 1% and 8%, which were significantly lower than the former (Fig. 4B). This result demonstrated that EPO enhanced cartilaginous callus formation during the early stage of bone healing.

EPO stimulates angiogenesis *in vitro* and *in vivo*

Besides the cartilage callus formation, angiogenesis is also a crucial factor during bone regeneration. To examine the role of EPO in angiogenesis, we isolated metatarsal bones from E17.5 mouse embryos and performed the endothelial sprouting assay in an *in vitro* culture system. As a result, the endothelial sprouting area was significantly increased by 1.9-fold in metatarsals treated with EPO 40 ng/ml compared with that of non-treatment controls, though this effect was not as dramatic as that of VEGF treatment (Fig. 5A and B). These results suggests that EPO may play a similar role like VEGF in stimulating endothelial sprouting from the metatarsal bones to form vessel like structure *in vitro*.

To further determine the effect of EPO in angiogenesis *in vivo*, we performed microCT angiography analysis in the middle stage of bone healing following local delivery of EPO at the fracture site. 3D reconstruction of newly formed vasculature at the fracture site showed more vascularity in the group treated with EPO than that of the control group (Fig. 5C). Microstructure parameters of the newly formed vessels including vessel volume (VV), vessel surface (VS) and vessel thickness (VTh) were all significantly increased following EPO administration compared with that of the control (Fig. 5D). And these changes were associated with the significantly enlarged total volume of callus area. These data demonstrated that EPO functioned as a proangiogenic factor during bone repair.

EPO enhances consolidation of bony callus during fracture healing

The observed positive effects of EPO on the cartilaginous callus formation and angiogenesis in the early and middle stages of bone healing led to an enhanced bone consolidation during facture healing. At day 28 post-surgery, the femurs from EPO treated group showed increased bone mass and density of the bony callus compared with the control group as evaluated by X-ray examination (Fig. 6A). X-ray score of the bony callus was increased by 1.5-fold in the EPO administrated group than that of the controls (Fig. 6B). Micro-CT 3D reconstruction of the bony callus clearly revealed a more robust bone formation in the EPO treated group than that of the controls (Fig. 6C), with the bone microstructure parameters of bone volume (BV), bone volume/total volume (BV/TV) and bone surface (BS) all significantly higher in the former than in the later (Fig. 6D). Further assessment of the biomechanical strength of the healing bones by three-point bending test showed that peak load, elastic modulus, bend strain at maximum, and bend strength at maximum were all significantly

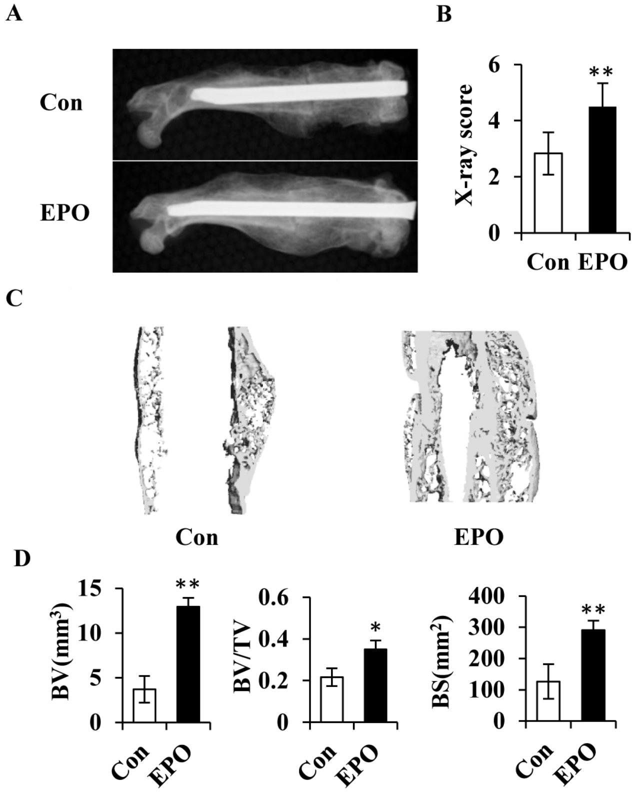

Figure 6. EPO increases the volume of the bony callus during consolidation of bone healing. (A) X-ray examination of the bony callus at day 28 post-surgery. Con, non-treatment control. (B) Quantitation of X-ray score of the bony callus at day 28 post-surgery. (C) Representative micro-CT 3D images of the new bone regenerates at day 28 post-surgery. (D) Quantitation of the bone microstructure parameters including bone volume (BV), bone volume/total volume (BV/TV), and bone surface (BS) by micro-CT analysis. *$P<0.05$; **$P<0.01$, $n=6$.
doi:10.1371/journal.pone.0102010.g006

increased in the group treated by EPO compared with the controls (Fig. 7A-D). Thus, local administration of EPO at the fracture site resulted in an increase in bony callus volume with improved biomechanical properties of the newly formed bone.

Discussion

Current procedures to promote skeletal regeneration include the applications of autografts, allografts, biocompatible implants, bioactive factors and mesenchymal stem cell-based therapy. However, the above approaches also face significant limitations due to insufficient supply, potential disease transmission, immuno-rejection, or less ability of functional engraftment. Thus, discovery of novel therapeutic approaches to improve skeletal repair and healing remains a great demand and clinical challenge in Orthopaedic regenerative medicine. In the present study, we demonstrated that local administration of EPO enhanced the consolidation and mechanical competence of the newly formed bone by promoting chondrogenic and angiogenic responses following injury, which can be explained by direct promotion effects of EPO on chondrocyte proliferation and differentiation, and on endothelial growth as shown in the *in vitro* studies. These findings expand the extent of EPO's tissue-specific functions and suggest that EPO may serve as a promising therapeutic agent for skeletal regeneration.

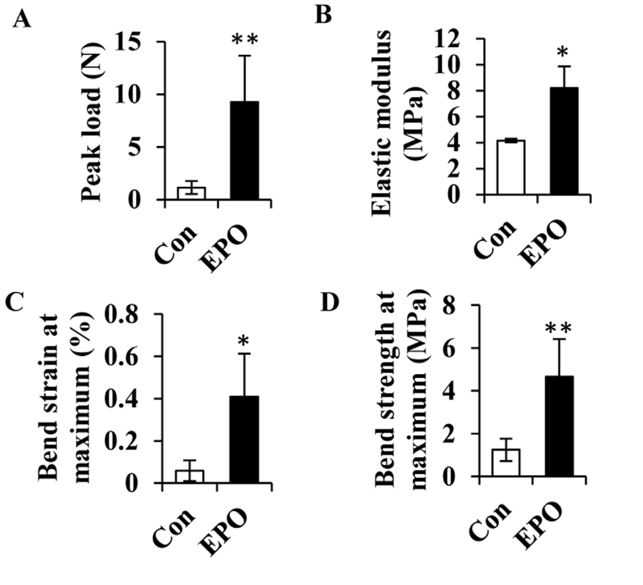

Figure 7. EPO improves biomechanical properties during consolidation of bone healing. Biomechanical parameters including peak load (A), elastic modulus (B), bend strain at maximum (C), and bend strength at maximum (D) were calculated at day 28 post-surgery. Con, non-treatment control. *$P<0.05$; **$P<0.01$, $n=3$.
doi:10.1371/journal.pone.0102010.g007

Previous study reported that EPOR was restrictedly expressed in hypertrophic chondrocytes during the cartilaginous callus stage of bone healing [45], and proposed that improved early endochondral ossification were mediated by EPO/EPOR signaling. In our study, knockdown of EPOR in primary chondrocytes resulted in a dramatic reduction of their responses to exogenous EPO in terms of cell proliferation (Fig. 2C) and chondrogenic marker genes expression upon chondrogenic differentiation (Fig. 3C). These evident reduction in EPO's effects on chondrocytes upon EPOR knockdown clearly demonstrated that EPO regulated the proliferation and differentiation of chondrocytes at least partially through EPOR.

Interestingly, EPO was also found to be present in chondrocytes in developing bones of new born mice as well as in newly formed cartilaginous callus of the healing bone in our study. Blockage of the endogenous EPO in primary chondrocytes using EPO block peptide impaired the chondrogenic differentiation, suggesting that endogenous EPO may function as an autocrine or paracrine factor for chondrocytes in normal bone development. Further clarifying the regulation of EPO production in chondrocytes in response to developmental and environmental cues will help better understanding its tissue-specific function in skeletal development and regeneration.

It is worthy to note that chondrocytes also produce VEGF [58,59], a well established angiogenic factor. Indeed, we observed a functional similarity of EPO and VEGF in the endothelial sprouting stimulation, indicating that both factors may exert overlapping but distinct roles in the angiogenic responses during bone healing. Given that EPO exerts versatile functions in varied tissues and cells and that bone regeneration is a complex process involving multiple cellular components or biological factors, the role of EPO in improving bone healing may also be versatile and complex. Direct and indirect signaling pathways may work coordinately to facilitate a better bone formation. For example, the increased volume of cartilaginous callus by EPO treatment may produce more VEGF and EPO to benefit angiogenesis and the increased vascularity by EPO administration may bring more

blood and nutrient supply and osteoprogenitor cells to promote the consolidation of the healing bone. In addition, EPO may function as a coupling factor to coordinate haematopoiesis and the homeostasis of the skeleton, which deserves to be furthur defined.

In summary, EPO and EPOR are expressed in the neonatal developing growth plate and cartilaginous callus of healing bone. EPO may function as an autocrine or paracrine factor to regulate the spatial and temporal coordination among angiogenesis, chondrogenesis and osteogenesis during bone repair. EPO may serve as an effective agent to promote skeletal regeneration.

Acknowledgments

We are grateful for the assistance of the Core laboratories at School of Biomedical Sciences, the Chinese University of Hong Kong, and Small Animal Phenotyping Core at the University of Alabama at Birmingham.

Author Contributions

Conceived and designed the experiments: CW LW. Performed the experiments: LW FZ QH WPT CW. Analyzed the data: LW CW FZ QH. Contributed reagents/materials/analysis tools: LL QL ZW GQ GZ. Contributed to the writing of the manuscript: CW LW.

References

1. Einhorn TA (1995) Enhancement of fracture-healing. J Bone Joint Surg Am 77: 940–956.
2. Solomon DH, Patrick AR, Schousboe J, Losina E (2014) The Potential Economic Benefits of Improved Post-Fracture Care: A Cost-Effectiveness Analysis of a Fracture Liaison Service in the US Health Care System. J Bone Miner Res.
3. Hak DJ, Fitzpatrick D, Bishop JA, Marsh JL, Tilp S, et al. (2014) Delayed union and nonunions: Epidemiology, clinical issues, and financial aspects. Injury 45 Suppl 2: S3-7.
4. Richards JB, Rivadeneira F, Inouye M, Pastinen TM, Soranzo N, et al. (2008) Bone mineral density, osteoporosis, and osteoporotic fractures: a genome-wide association study. Lancet 371: 1505–1512.
5. Janghorbani M, Van Dam RM, Willett WC, Hu FB (2007) Systematic review of type 1 and type 2 diabetes mellitus and risk of fracture. Am J Epidemiol 166: 495–505.
6. Siddiqui NA, Owen JM (2013) Clinical advances in bone regeneration. Curr Stem Cell Res Ther 8: 192–200.
7. Wan C, Gilbert SR, Wang Y, Cao X, Shen X, et al. (2008) Activation of the hypoxia-inducible factor-1alpha pathway accelerates bone regeneration. Proc Natl Acad Sci U S A 105: 686–691.
8. Fassbender M, Strobel C, Rauhe JS, Bergmann C, Schmidmaier G, et al. (2011) Local inhibition of angiogenesis results in an atrophic non-union in a rat osteotomy model. Eur Cell Mater 22: 1–11.
9. Ferguson C, Alpern E, Miclau T, Helms JA (1999) Does adult fracture repair recapitulate embryonic skeletal formation? Mech Dev 87: 57–66.
10. Gerstenfeld LC, Cullinane DM, Barnes GL, Graves DT, Einhorn TA (2003) Fracture healing as a post-natal developmental process: molecular, spatial, and temporal aspects of its regulation. J Cell Biochem 88: 873–884.
11. Kanczler JM, Oreffo RO (2008) Osteogenesis and angiogenesis: the potential for engineering bone. Eur Cell Mater 15: 100–114.
12. Brandi ML (2013) Healing of the bone with anti-fracture drugs. Expert Opin Pharmacother 14: 1441–1447.
13. McKibbin B (1978) The biology of fracture healing in long bones. J Bone Joint Surg Br 60-B: 150–162.
14. Kolar P, Gaber T, Perka C, Duda GN, Buttgereit F (2011) Human early fracture hematoma is characterized by inflammation and hypoxia. Clin Orthop Relat Res 469: 3118–3126.
15. Sun H, Jung Y, Shiozawa Y, Taichman RS, Krebsbach PH (2012) Erythropoietin modulates the structure of bone morphogenetic protein 2-engineered cranial bone. Tissue Eng Part A 18: 2095–2105.
16. Kigami R, Sato S, Tsuchiya N, Yoshimakai T, Arai Y, et al. (2013) FGF-2 angiogenesis in bone regeneration within critical-sized bone defects in rat calvaria. Implant Dent 22: 422–427.
17. Warren SM, Steinbrech DS, Mehrara BJ, Saadeh PB, Greenwald JA, et al. (2001) Hypoxia regulates osteoblast gene expression. J Surg Res 99: 147–155.
18. Sheng MH, Lau KH, Baylink DJ (2014) Role of Osteocyte-derived Insulin-Like Growth Factor I in Developmental Growth, Modeling, Remodeling, and Regeneration of the Bone. J Bone Metab 21: 41–54.
19. Zelzer E, McLean W, Ng YS, Fukai N, Reginato AM, et al. (2002) Skeletal defects in VEGF(120/120) mice reveal multiple roles for VEGF in skeletogenesis. Development 129: 1893–1904.
20. Street J, Bao M, deGuzman L, Bunting S, Peale FV Jr, et al. (2002) Vascular endothelial growth factor stimulates bone repair by promoting angiogenesis and bone turnover. Proc Natl Acad Sci U S A 99: 9656–9661.
21. Wang Y, Wan C, Deng L, Liu X, Cao X, et al. (2007) The hypoxia-inducible factor alpha pathway couples angiogenesis to osteogenesis during skeletal development. J Clin Invest 117: 1616–1626.
22. Wan C, Shao J, Gilbert SR, Riddle RC, Long F, et al. (2010) Role of HIF-1alpha in skeletal development. Ann N Y Acad Sci 1192: 322–326.
23. Wu H, Liu X, Jaenisch R, Lodish HF (1995) Generation of committed erythroid BFU-E and CFU-E progenitors does not require erythropoietin or the erythropoietin receptor. Cell 83: 59–67.
24. Wu H, Klingmuller U, Besmer P, Lodish HF (1995) Interaction of the erythropoietin and stem-cell-factor receptors. Nature 377: 242–246.
25. Kertesz N, Wu J, Chen TH, Sucov HM, Wu H (2004) The role of erythropoietin in regulating angiogenesis. Dev Biol 276: 101–110.
26. Brines M, Grasso G, Fiordaliso F, Sfacteria A, Ghezzi P, et al. (2004) Erythropoietin mediates tissue protection through an erythropoietin and common beta-subunit heteroreceptor. Proc Natl Acad Sci U S A 101: 14907–14912.
27. Brines M, Cerami A (2006) Discovering erythropoietin's extra-hematopoietic functions: biology and clinical promise. Kidney Int 70: 246–250.
28. Tan CC, Eckardt KU, Ratcliffe PJ (1991) Organ distribution of erythropoietin messenger RNA in normal and uremic rats. Kidney Int 40: 69–76.
29. Fisher JW (1997) Erythropoietin: physiologic and pharmacologic aspects. Proc Soc Exp Biol Med 216: 358–369.
30. Yi C, Hak DJ (2012) Traumatic spinopelvic dissociation or U-shaped sacral fracture: a review of the literature. Injury 43: 402–408.
31. Tada H, Kagaya Y, Takeda M, Ohta J, Asaumi Y, et al. (2006) Endogenous erythropoietin system in non-hematopoietic lineage cells plays a protective role in myocardial ischemia/reperfusion. Cardiovasc Res 71: 466–477.
32. Kumral A, Tuzun F, Oner MG, Genc S, Duman N, et al. (2011) Erythropoietin in neonatal brain protection: the past, the present and the future. Brain Dev 33: 632–643.
33. Tsai PT, Ohab JJ, Kertesz N, Groszer M, Matter C, et al. (2006) A critical role of erythropoietin receptor in neurogenesis and post-stroke recovery. J Neurosci 26: 1269–1274.
34. Hak DJ, Makino T, Niikura T, Hazelwood SJ, Curtiss S, et al. (2006) Recombinant human BMP-7 effectively prevents non-union in both young and old rats. J Orthop Res 24: 11–20.
35. Acheson A, Richards JB, de Wit H (2007) Effects of sleep deprivation on impulsive behaviors in men and women. Physiol Behav 91: 579–587.
36. Cianferotti L, Brandi ML (2014) Muscle-bone interactions: basic and clinical aspects. Endocrine 45: 165–177.
37. Wright GL, Hanlon P, Amin K, Steenbergen C, Murphy E, et al. (2004) Erythropoietin receptor expression in adult rat cardiomyocytes is associated with an acute cardioprotective effect for recombinant erythropoietin during ischemia-reperfusion injury. FASEB J 18: 1031–1033.
38. Bernaudin M, Marti HH, Roussel S, Divoux D, Nouvelot A, et al. (1999) A potential role for erythropoietin in focal permanent cerebral ischemia in mice. J Cereb Blood Flow Metab 19: 643–651.
39. Sugawa M, Sakurai Y, Ishikawa-Ieda Y, Suzuki H, Asou H (2002) Effects of erythropoietin on glial cell development; oligodendrocyte maturation and astrocyte proliferation. Neurosci Res 44: 391–403.
40. Lee MY, Fukunaga R, Lee TJ, Lottsfeldt JL, Nagata S (1991) Bone modulation in sustained hematopoietic stimulation in mice. Blood 77: 2135–2141.
41. Takenaka T, Itaya Y, Ishikawa I, Kobayashi K, Tsuchiya Y (2003) Skeletal effects of erythropoietin in hemodialysis patients. Int Urol Nephrol 35: 407–413.
42. Singbrant S, Russell MR, Jovic T, Liddicoat B, Izon DJ, et al. (2011) Erythropoietin couples erythropoiesis, B-lymphopoiesis, and bone homeostasis within the bone marrow microenvironment. Blood 117: 5631–5642.
43. Shiozawa Y, Jung Y, Ziegler AM, Pedersen EA, Wang J, et al. (2010) Erythropoietin couples hematopoiesis with bone formation. PLoS One 5: e10853.
44. Garcia P, Speidel V, Scheuer C, Laschke MW, Holstein JH, et al. (2011) Low dose erythropoietin stimulates bone healing in mice. J Orthop Res 29: 165–172.
45. Holstein JH, Menger MD, Scheuer C, Meier C, Culemann U, et al. (2007) Erythropoietin (EPO): EPO-receptor signaling improves early endochondral ossification and mechanical strength in fracture healing. Life Sci 80: 893–900.
46. Mihmanli A, Dolanmaz D, Avunduk MC, Erdemli E (2009) Effects of recombinant human erythropoietin on mandibular distraction osteogenesis. J Oral Maxillofac Surg 67: 2337–2343.
47. Gosset M, Berenbaum F, Thirion S, Jacques C (2008) Primary culture and phenotyping of murine chondrocytes. Nat Protoc 3: 1253–1260.
48. Derks M, Sturm T, Haverich A, Hilfiker A (2013) Isolation and chondrogenic differentiation of porcine perichondrial progenitor cells for the purpose of cartilage tissue engineering. Cells Tissues Organs 198(3):179–189.
49. Ovchinnikov D (2009) Alcian blue/alizarin red staining of cartilage and bone in mouse. Cold Spring Harb Protoc 2009: pdb prot5170.
50. Lee YJ, Kong MH, Song KY, Lee KH, Heo SH (2008) The Relation Between Sox9, TGF-beta1, and Proteoglycan in Human Intervertebral Disc Cells. J Korean Neurosurg Soc 43: 149–154.

51. Enobakhare BO, Bader DL, Lee DA (1996) Quantification of sulfated glycosaminoglycans in chondrocyte/alginate cultures, by use of 1,9-dimethylmethylene blue. Anal Biochem 243: 189–191.
52. Deckers M, van der Pluijm G, Dooijewaard S, Kroon M, van Hinsbergh V, et al. (2001) Effect of angiogenic and antiangiogenic compounds on the outgrowth of capillary structures from fetal mouse bone explants. Lab Invest 81: 5–15.
53. Cackowski FC, Anderson JL, Patrene KD, Choksi RJ, Shapiro SD, et al. (2010) Osteoclasts are important for bone angiogenesis. Blood 115: 140–149.
54. Lane JM, Sandhu HS (1987) Current approaches to experimental bone grafting. Orthop Clin North Am 18: 213–225.
55. Casazza K, Hanks LJ, Clines GA, Tse HM, Eberhardt AW (2013) Diabetes-related impairment in bone strength is established early in the life course. World J Diabetes 4: 145–150.
56. Akiyama H, Lefebvre V (2011) Unraveling the transcriptional regulatory machinery in chondrogenesis. J Bone Miner Metab 29: 390–395.
57. Akiyama H, Chaboissier MC, Martin JF, Schedl A, de Crombrugghe B (2002) The transcription factor Sox9 has essential roles in successive steps of the chondrocyte differentiation pathway and is required for expression of Sox5 and Sox6. Genes Dev 16: 2813–2828.
58. Haigh JJ, Gerber HP, Ferrara N, Wagner EF (2000) Conditional inactivation of VEGF-A in areas of collagen2a1 expression results in embryonic lethality in the heterozygous state. Development 127: 1445–1453.
59. De Spiegelaere W, Cornillie P, Van den Broeck W (2010) Localization of erythropoietin in and around growing cartilage. Mol Cell Biochem 337: 287–291.
